# Corrosion Inhibition in Concrete: Synergistic Performance of Hybrid Steel-Polypropylene Fiber Reinforcement Against Marine Salt Spray

**DOI:** 10.3390/polym17192645

**Published:** 2025-09-30

**Authors:** Jianqiao Yu, Jamal A. Abdalla, Rami A. Hawileh, Xiaoyue Zhang, Zhigang Zhang

**Affiliations:** 1 School of Civil Engineering, Chongqing University, Chongqing 400045, China; 2 Department of Civil Engineering, American University of Sharjah, Sharjah P.O. Box 26666, United Arab Emirates; 3 State Key Laboratory of Safety and Resilience of Civil Engineering in Mountain Area, Chongqing University, Chongqing 400045, China

**Keywords:** fiber reinforced concrete, steel fiber, PP fiber, salt spray, chloride corrosion

## Abstract

In the marine salt spray environment, steel fiber reinforced concrete (SFRC) structures are often subjected to accelerated durability degradation, primarily due to chloride-induced corrosion. To address this issue, polypropylene (PP) fibers were incorporated to partially replace steel fibers in the formulation of hybrid fiber reinforced concrete (HFRC), thereby enhancing its resistance to chloride corrosion. The results demonstrate that all HFRC mixtures achieved a compressive strength of approximately 65 MPa at 28 d. After 200 d of salt spray exposure, the compressive strength of the HFRC containing PP fibers decreased at a significantly slower rate than that of the control group (M0) incorporating sole steel fibers, with the former still meeting the high-strength concrete standard (>60 MPa). Regardless of the exposure duration to salt spray, the wave velocity of the HF series remained higher than that of M0. This suggests that the PP fibers play a significant role in preserving the matrix’s compactness, effectively mitigating deterioration caused by chloride corrosion. Furthermore, after 200 d of exposure, the peak chloride content, critical corrosion depth, and chloride diffusion coefficient of HF2 were 0.58%, 16 mm, and 1.24 × 10^−12^ m^2^/s, respectively, all of which were lower than those of the other specimens. This demonstrates that incorporating 0.3 vol% PP fibers most effectively enhances the chloride corrosion resistance of HFRC.

## 1. Introduction

Concrete has become an indispensable material in construction due to its abundant availability of raw materials, relatively low cost, and exceptional compressive strength. However, its inherent brittleness, low toughness, and susceptibility to cracking pose significant challenges, making concrete structures susceptible to premature deterioration from mechanical and environmental loads, thereby compromising structural integrity [1,2,3]. Since concrete is intrinsically a multi-scale composite material composed of a cementitious matrix and aggregates, the theoretical incorporation of specific fiber types can enhance its mechanical properties, thereby meeting the stricter demands of critical structures [4,5].

Steel fiber reinforced concrete (SFRC) is a typical fiber-reinforced composite material, formed by incorporating short steel fibers into ordinary concrete. The addition of steel fibers facilitates the transfer of load-induced stresses to the surrounding concrete matrix near cracks [6], converting brittle failure into ductile failure [5,7], thereby significantly enhancing the crack resistance of the concrete. Moreover, SFRC exhibits exceptional toughness, retaining significant residual strength even after the formation of the first crack [8]. As a result of its superior properties, SFRC has seen significant advancements over the past decade, substantially expanding the potential range of its applications [9,10].

However, with the escalating utilization of marine resources, an expanding range of infrastructures is being constructed in marine environments, such as dry docks, seawalls, ports, trans-oceanic bridges, and artificial islands. Therefore, the durability of SFRC is significantly threatened. The evaporation of seawater results in the accumulation of a significant quantity of salt spray in the atmosphere [11]. When SFRC mixtures are subjected to such corrosive environments, chlorides in the salt spray persistently accumulate on the concrete surface, progressively penetrating the interior through the combined effects of diffusion and capillary adsorption [11,12,13]. The high-humidity property of marine environments accelerates the diffusion of chloride ions, leading to a deterioration in material durability and a reduction in the structure’s service life.

In recent years, significant research has focused on evaluating the durability performance of SFRC mixtures under chloride exposure conditions. For instance, Balouch et al. [14] found that SFRC mixtures not only exhibited clear signs of corrosion under chloride exposure but also suffered a substantial deterioration in durability. Hwang et al. [15] further reported that the corrosion of steel fibers substantially reduced the interfacial bond strength between the fibers and the surrounding matrix, thereby undermining the load-bearing capacity and overall safety of the SFRC structure. Pyo et al. [16] indicated that the considerable presence of steel fibers in the concrete forms conductive pathways, facilitating electrochemical corrosion of SFRC when exposed to chloride solutions. Their study revealed that SFRC mixtures, after immersion in a 10% chloride solution for 180 d, exhibited a 10% reduction in both flexural strength and toughness. In conclusion, these studies collectively underscore a significant decline in the durability of SFRC mixtures in chloride conditions. Consequently, advancing research to improve the chloride corrosion resistance of SFRC mixtures has become an urgent priority.

To mitigate the detrimental effects of chlorides, the inhibition of chloride ion transport during the corrosion process is vital for enhancing the corrosion resistance of concrete. Based on the previous literature, it is widely recognized that incorporating polypropylene (PP) fibers can effectively mitigate microcrack expansion, thereby reducing the rate of chloride penetration, eventually enhancing the concrete’s resistance to corrosion. For example, Chen et al. [17] highlighted that PP fibers play a crucial role in controlling crack initiation and propagation, a key mechanism for preventing chloride penetration, especially during the early stages of chloride exposure in concrete. Moreover, Afroughsabet et al. [18] found that incorporating 0.15% to 0.45% of PP fibers significantly enhances the concrete’s resistance to chloride penetration. The addition of PP fibers reduces the porosity of the concrete and improves its internal structure, resulting in a decrease in the chloride diffusion coefficient by up to 78%. Furthermore, Moradi et al. [19] conducted electrochemical analysis to investigate the corrosion behavior of PP fibers in concrete, demonstrating that PP fiber reinforced concrete (PFRC) offers significantly greater resistance to chloride corrosion than conventional concrete. Additionally, Singh et al. [20] demonstrated that the co-incorporation of steel and PP fibers into concrete offers significant benefits. The distinct crack-resisting mechanisms of these two fiber types enable them to complement one another, thereby synergistically enhancing the mechanical properties and durability of the concrete structure [21,22,23]. In light of the above, partially replacing steel fibers with PP fibers presents a viable and effective strategy for enhancing the chloride corrosion resistance of SFRC mixtures.

Following prior studies, this research argues that the incorporation of PP fibers to partially replace steel fibers in the formulation of hybrid fiber reinforced concrete (HFRC) offers a promising strategy for mitigating durability degradation caused by chloride corrosion. Regrettably, current research on HFRC mixtures predominantly focuses on the mechanical properties, while the long-term effects of HFRC in chloride-rich conditions remain insufficiently explored. Furthermore, the precise mechanisms by which the hybrid incorporation of fibers enhances resistance to chloride corrosion remain unclear, thus limiting the broader application of these systems. Considering the synergy between the fiber-matrix bond, pore structure, and fiber dispersion, the incorporation of PP fibers enhances the crack resistance of the matrix and reduces its permeability, both of which are crucial for improving the durability of salt-resilient marine concrete [24,25]. Moreover, the fine diameter of PP fibers can effectively mitigate microcracks caused by chloride penetration, thus reducing the transmission pathways for chloride ion transmission. These mechanisms hold promise for enhancing the chloride corrosion resistance of HFRC mixtures.

Based on the aforementioned conditions, in this paper, the HFRC mixtures were developed by incorporating PP fibers as partial substitutes for the steel fibers, thereby enhancing the resistance to chloride corrosion. In the following sections, firstly, the compressive strength of the HFRC mixtures was investigated to evaluate the potential of PP fibers in mitigating the reduction in load-bearing capacity of HFRC under salt spray conditions. Subsequently, the ultrasonic wave test was utilized to examine the effect of salt spray corrosion on the microstructural properties of HFRC mixtures. Finally, the chloride content test was conducted to explore the changes in peak chloride content, the critical corrosion depth, and the chloride diffusion coefficient with respect to the corrosion time. Meanwhile, the role of PP fibers in mitigating chloride penetration into HFRC was discussed. The novelty of this study lies in its in-depth examination of the deterioration mechanisms of HFRC mixtures in chloride conditions, with a particular emphasis on the fiber-matrix interfacial transition zone (ITZ). This research offers new insights into the role of PP fibers in enhancing the HFRC’s resistance to chloride corrosion at the microscopic level. As a result, it addresses a significant gap in current knowledge and contributes to the advancement of HFRC in marine engineering applications.

## 2. Experimental Programs

### 2.1. Materials and Specimen Preparations

In this study, the HFRC mixture consisted of cementitious binders, coarse aggregates (CA), fine aggregates, water (W), superplasticizer (SP), steel fibers, and polypropylene (PP) fibers. The binder materials included P.O. 42.5 Ordinary Portland Cement (C), Class II fly ash (FA), and silica fume (SF). The chemical composition of the cementitious materials, as analyzed by X-ray fluorescence (XRF), is summarized in Table 1.

To optimize the workability and flowability of the HFRC matrix, the coarse aggregate was selected to be ellipsoidal crushed stone with a crushing index of 7%, and a particle size distribution ranging from 5 to 20 mm; the fine aggregate consisted of silica sand (SS), with an apparent density of 2600 kg/m^3^ and a fineness modulus of 2.7.

**Figure 1:**
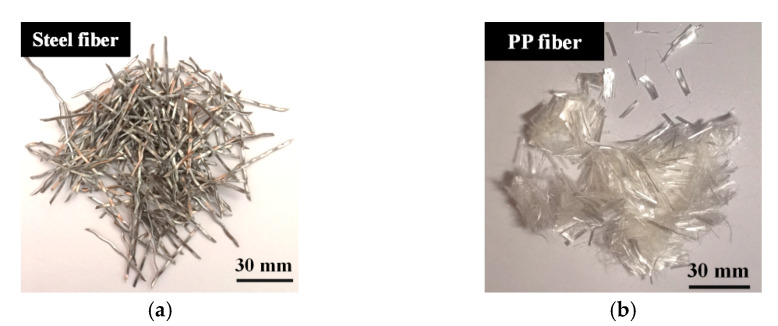
The appearances of steel fiber and PP fiber: (**a**) steel fiber; (**b**) PP fiber.

The steel fibers and PP fibers in the HFRC are depicted in Figure 1. Table 2 presents the physical and mechanical properties of the two types of fibers, as provided by the manufacturers.

The steel fibers are 40 mm in length and 0.30 mm in diameter. They exhibit excellent mechanical properties, with a remarkable elastic modulus of 200 GPa and a tensile strength of 1270 MPa. However, the elongation of the steel fibers is limited to 5%, indicating a relatively low capacity for deformation before failure.

In contrast, the PP fibers are notably finer and shorter, with a length of 12 mm and a diameter of 0.03 mm. Both the elastic modulus (4.8 GPa) and tensile strength (500 MPa) of PP fibers are lower than those of steel fibers. However, PP fibers are distinguished by their high elongation of 17%, which enables them to undergo significant deformation before failure, thus enhancing energy absorption and effectively preventing the propagation of microcracks [26]. Furthermore, with a density of 910 kg/m^3^, PP fibers are considerably lighter than steel fibers, making them particularly advantageous in applications where weight reduction is a crucial factor [27].

Table 3 details the HFRC mix proportions applied in this research. The mixtures consisted of two series, designed to include HFRC with different fiber types and contents. Specifically, based on previous research [28], a 2% volume fraction of steel fibers was selected to prepare the SFRC mixture (named as M0), which served as the control group. In the experimental series, PP fibers were replaced with steel fibers at volume fractions of 0.1%, 0.3%, and 0.5%, resulting in the formation of HFRC mixtures, named as HF1, HF2, and HF3, respectively. The selection of a maximum PP fiber content of 0.5% was based on the difficulty in dispersing higher amounts of PP fibers, as higher content tends to hinder proper mixing [29]. In all mixtures, a water-to-binder ratio of 0.32 and a silica fume-to-cement ratio of 0.05 were adopted to formulate the high-strength concrete matrix. A dosage of 4 kg/m^3^ of SP was employed to ensure optimal workability and mixing efficiency.

**Figure 2:**
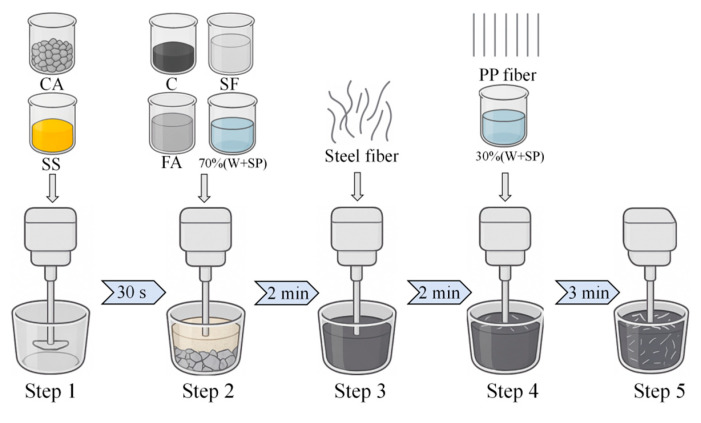
Schematic diagram of the mixing procedure for HFRC.

The HFRC mixtures were prepared through a series of meticulously controlled mixing steps to ensure uniformity and consistent performance. The mixing procedure for the HFRC mixture is illustrated in Figure 2. To begin with, SS and CA were introduced into the mixer and dry mixed for 30 s. Following this, all cementitious materials (C+FA+SF) and a mixed solution (W+SP) of 70% were added and mixed for 2 min, forming a consistent and uniform HFRC paste. Subsequently, the steel fibers were incorporated and mixed for 2 min to ensure even dispersion throughout the HFRC paste. Finally, the PP fibers and the remaining 30% of the mixed solution were added and mixed for 3 min until a homogenous dispersion was achieved. The fresh paste exhibited exceptional flowability, allowing for easy pouring into molds with dimensions of 100 mm × 100 mm × 100 mm after 3 min of vibration. After 24 h of curing, the specimens were demolded and cured at 25 ± 3 °C temperature and 55 ± 5% relative humidity for 28 d before testing.

### 2.2. Testing Procedures

**Figure 3:**
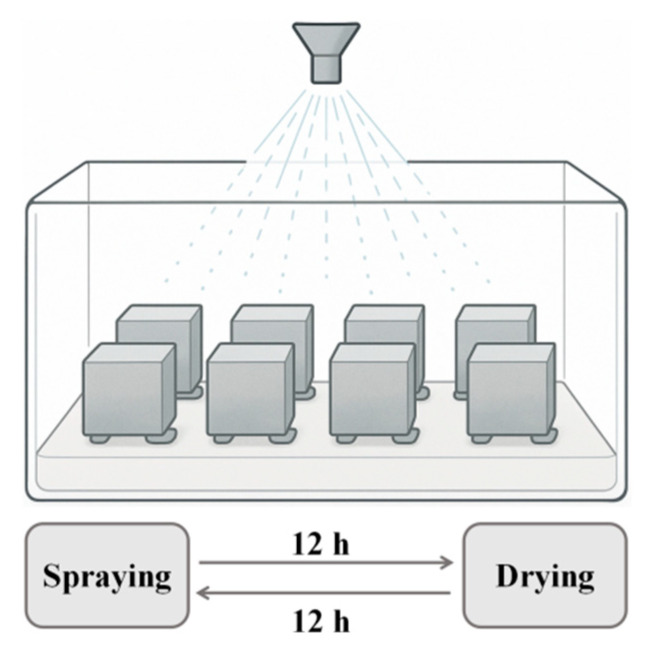
Schematic diagram of salt spray test.

Salt spray test was conducted in accordance with ASTM B117 [30] to simulate the corrosion environment of HFRC mixtures in marine conditions. The detailed experimental procedure is presented in Figure 3. Prior to the test, five surfaces of all HFRC mixtures were sealed with epoxy resin, leaving only one surface exposed to the corrosion environment, thus ensuring unidirectional chloride ion transfer. To expedite the corrosion process, an intermittent spraying method was employed, with a 12 h spraying period followed by 12 h of natural drying. Each cycle lasted 24 h, with a total of 200 cycles conducted, which is approximately equivalent to 2 years of marine exposure based on typical corrosion rates observed in marine environments [12,13]. The salt spray consisted of a 3.5% NaCl solution, with a deposition rate of 2 mL/h·cm^2^. The internal temperature and humidity of the salt spray chamber were maintained at 25 °C and 85%, respectively.

Compressive strength tests were conducted according to the GB/T 50081-2019 [31] to investigate the mechanical properties of HFRC in a corrosion environment. The specimens measuring 100 mm × 100 mm × 100 mm were exposed to a salt spray corrosion environment for 0, 50, 100, 150, and 200 d before the testing was performed. The loading was applied at a rate of 1.0 MPa/s until failure, and the maximum load at fracture was recorded to calculate the final result. For each mixture, the reported compressive strength represents the average value obtained from three replicate specimens tested under identical conditions.

Mercury intrusion porosimetry (MIP) tests were conducted in accordance with ASTM D4404 [32], with the objective of investigating the influence of salt spray corrosion on the microstructures of HFRC mixtures. During sample preparation, fragments approximately 1 cm in size were extracted from the centers of HFRC cubic specimens, which had been exposed to a salt spray environment for 0 and 200 d, and subsequently immersed in anhydrous ethanol to terminate hydration. Prior to testing, the fragments were dried at 60 °C until a constant mass was achieved. The applied working pressure for this test was 414 MPa, facilitating the measurement of pore sizes spanning from 6 to 800,000 nm.

**Figure 4:**
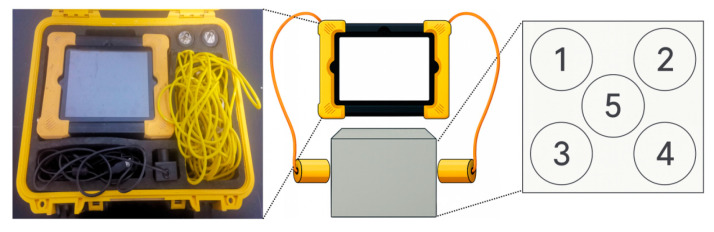
Ultrasonic test setup and distribution of testing points.

Ultrasonic tests were carried out in compliance with CECS 02:2005 [33], aiming to perform non-destructive evaluation of the chloride ingress within the HFRC mixtures. Prior to testing, a thin layer of petroleum jelly was applied to the testing surface of the specimens to mitigate air interference between the concrete samples and the sensor interface. The specific testing procedure and measurement locations are depicted in Figure 4. The ultrasonic waves propagated longitudinally along the specimens, with each specimen subjected to five separate tests, from which the average velocity was taken as the final result. This approach has been previously applied in studies investigating the matrix compactness in fiber-reinforced concrete [34,35].

Scanning electron microscope (SEM) analyses were employed to investigate the alterations in the micromorphology of HFRC mixtures subjected to different exposure times in salt spray environments. The test samples were crushed and sieved to obtain mortar fragments with dimensions ranging from 3 to 5 mm. These fragments were then immersed in anhydrous ethanol for 24 h to terminate hydration, after which they were dried to a constant weight at 60 °C. During the examination, the sample was securely mounted on the base using double-sided conductive adhesive, followed by a gold sputter coating to enhance conductivity.

**Figure 5:**
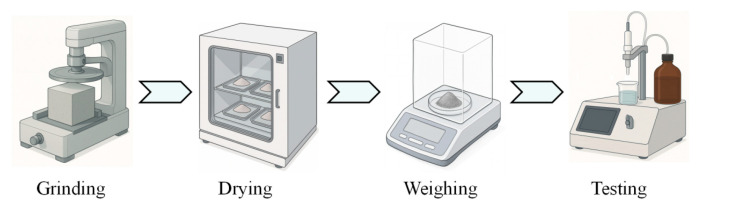
Test procedure for chloride content.

Chloride content tests were conducted in accordance with GB/T 50476-2019 [36], with the aim of analyzing parameters related to chloride corrosion in HFRC mixtures under salt spray conditions. The test procedure is illustrated in Figure 5. To begin with, surface contaminants from the specimen, resulting from the salt spray environment, were removed. A concrete grinding machine was employed to grind the specimen layer by layer. Given that the surface layer had experienced significant chloride corrosion, grinding was performed at 1 mm intervals for the 1–10 mm depth, while the remaining portion was ground at 2 mm intervals to a depth of 20 mm. The collected powder was then sieved using a 30-mesh fine sieve. Subsequently, the powder samples were dried at 60 °C for 24 h to remove residual moisture. Finally, the dried powder was weighed to 5 g, immersed in 100 mL of deionized water, and thoroughly dispersed. The chloride content was then measured using a rapid chloride ion content analyzer, as described in Ref. [13].

## 3. Results and Discussion

### 3.1. Compressive Strength and Mass Loss

**Figure 6:**
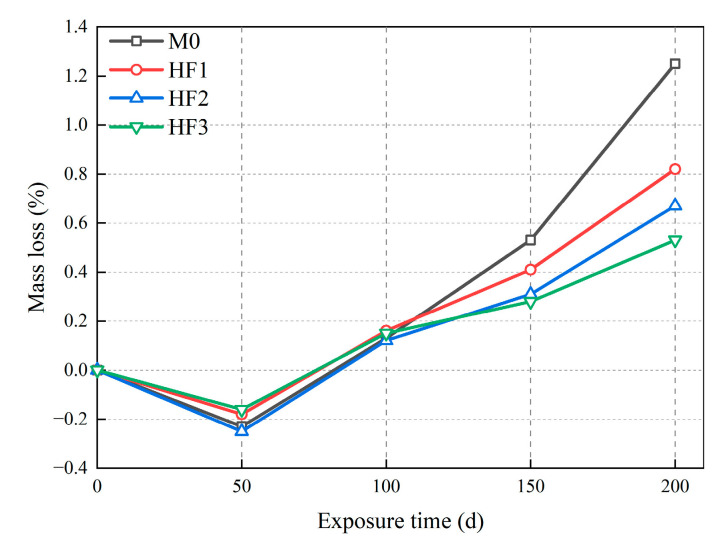
Mass loss of HFRC mixtures with exposure time under salt spray conditions.

Figure 6 illustrates the relationship between the mass loss of HFRC mixtures and exposure time under salt spray conditions. As displayed in Figure 6, the mass of the mixtures shows an increase at first, followed by a decrease as the exposure time progresses. In the first stage, from 0 to 50 d, the mass of all mixtures increased by 0.16% to 0.25%. This is primarily due to the continued hydration reaction of the cement matrix in the early stages of salt spray corrosion, leading to a denser cementitious structure [37]. However, as the exposure time extends from 50 to 100 d, the mass of all HFRC mixtures begins to decrease. At this point, the damage caused by chloride corrosion surpasses the compensatory effects of the hydration reaction [38]. Notably, after 150 d of exposure, the mass loss of the control group (M0) begins to exceed that of the HF series. Ultimately, after 200 d of exposure, the mass loss of M0 reaches 1.25%, significantly surpassing that of HF1 (0.82%), HF2 (0.67%), and HF3 (0.53%). This difference may be due to the fact that, unlike PP fibers, steel fibers provide a conductive pathway for chloride ions [39]. Prolonged exposure to a wet-dry salt spray environment in SFRC induces electrochemical corrosion, resulting in the formation of internal cracks within the matrix [40]. Consequently, the propagation of internal cracks leads to surface spalling, thereby resulting in a relatively larger mass loss for M0.

**Figure 7:**
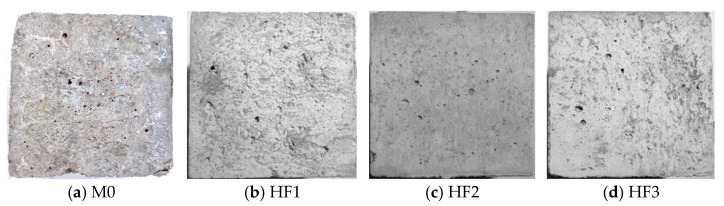
Apparent damage of HFRC mixtures after salt spray test.

Figure 7 displays the appearance of HFRC mixtures at the completion of the salt spray test. From Figure 7, it is evident that after 200 d of exposure, the M0 exhibits the most significant damage, with noticeable surface spalling. In contrast, the damage in HF1, HF2, and HF3 is comparatively minor, with no visible deterioration on the surface. This suggests that the incorporation of PP fibers into the SFRC mixture significantly mitigates the mass loss induced by the salt spray exposure. This effect is attributed to the ability of PP fibers to effectively link the microcracks formed due to chloride corrosion, thereby inhibiting microcrack propagation, which in turn slows the progression of surface spalling.

**Figure 8:**
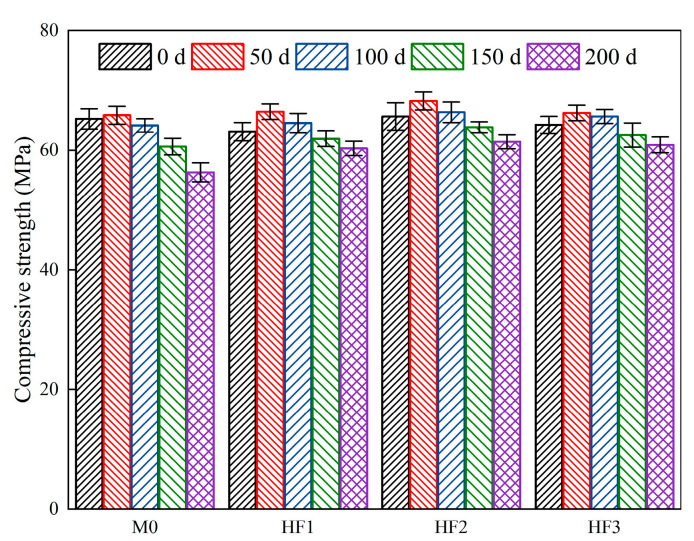
Compressive strength of HFRC mixtures with different exposure times.

Figure 8 presents the compressive strength of HFRC mixtures at varying exposure times under salt spray conditions. At 0 d, the compressive strength of M0 is 65.2 MPa. Similarly to the trend observed in mass loss, at 50 d of exposure, M0 exhibits a slight increase in the compressive strength, rising to 65.8 MPa. However, upon reaching 100 d, a slight decrease is observed, with the compressive strength reducing to 64.1 MPa. When the exposure time exceeds 150 d, a more pronounced decline is evident. Specifically, at 150 d of exposure, the compressive strength of M0 drops to 60.6 MPa, and at 200 d, it further decreases to 56.3 MPa, representing reductions of 7.06% and 13.65%, respectively, compared to the value at 0 d.

For HFRC mixtures containing PP fibers, the compressive strength of HF1, HF2, and HF3 prior to testing is comparable to that of M0, measuring 63.1 MPa, 65.6 MPa, and 64.2 MPa, respectively. Upon exposure to 50 d, the compressive strength growth rate of the HF series is similar to that of M0. At 100 d, the compressive strength of the HF series experiences a slight decline, though it remains higher than the original strength. Notably, after 150 d of exposure, the compressive strength of the HFRC mixtures, due to the presence of PP fibers, consistently surpasses that of M0. Specifically, the compressive strength of HF1, HF2, and HF3 declines to 61.9 MPa, 63.8 MPa, and 62.5 MPa, respectively, representing increases of 2.14%, 5.28%, and 3.14% compared to M0. At 200 d of exposure, the compressive strength of HF1, HF2, and HF3 is 60.3 MPa, 61.4 MPa, and 60.9 MPa, still exceeding the 60 MPa benchmark for high-strength concrete [41]. Therefore, the HFRC mixtures produced in this study demonstrate a potential for exceptional durability.

**Figure 9:**
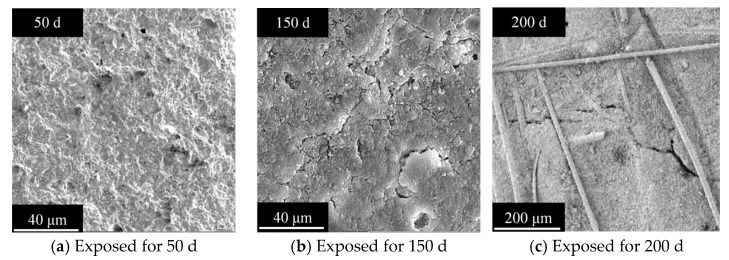
Representative microstructures of HFRC after salt spray conditions (exemplified by HF2 as a representative case).

Figure 9 shows the microstructure of HFRC following salt spray corrosion, which indirectly reveals the variation in compressive strength. As shown in Figure 9a, after 50 d of exposure, the continued hydration of the cementitious materials dominates over chloride-induced degradation, resulting in a well-preserved matrix with a dense structure. However, after 150 d of exposure, as observed in Figure 9b, a significant number of microcracks form within the HFRC matrix. These microcracks are attributed to the accumulation of expansive stresses caused by chlorides during the wet-dry cycles. The propagation of these microcracks leads to the spalling of the cement paste, which is macroscopically reflected in the decline of compressive strength. Figure 9c provides compelling evidence, indicating that the incorporation of PP fibers into HFRC effectively interrupts the propagation of microcracks after 200 d of salt spray exposure. All of these observations provide a coherent explanation for the variation in compressive strength of the HFRC mixtures during salt spray corrosion, particularly highlighting the role of PP fibers in mitigating microcrack propagation, which significantly slows down the reduction in compressive strength and enhances long-term durability.

**Figure 10:**
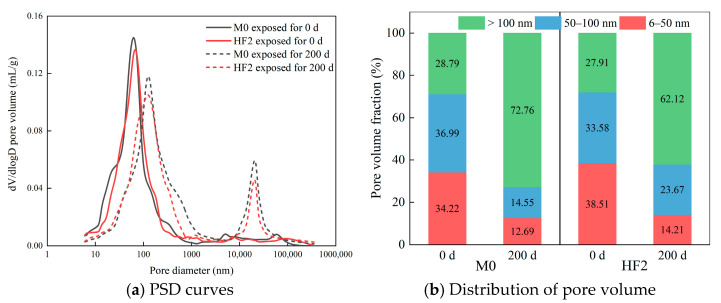
Distribution of pore diameters in HFRC mixtures.

To further investigate the effect of salt spray corrosion on the compressive strength of HFRC mixtures, a comparative analysis of the pore structures between M0 and HF2 was conducted. Figure 10 depicts the pore size distribution (PSD) and pore volume fractions of M0 and HF2 after 0 d and 200 d of salt spray exposure. Based on the classification of pore sizes proposed by Metha et al. [42], the pores are categorized into three distinct types: meso-pores (4.5–50 nm), middle capillary pores (50–100 nm), and large capillary pores (>100 nm). Notably, the large capillary pores exert a significant influence on the mechanical properties and durability of concrete.

From Figure 10a, it is evident that before the salt spray testing, the peak height of HF2 in the 60–70 nm range is slightly lower than that of M0, suggesting that HF2 possesses a lower pore volume within this range. This phenomenon can be partly attributed to the incorporation of PP fibers, which fill larger pores and connect defects within the matrix, thereby promoting the transformation of the middle capillary pores of the HFRC into mesopores [43]. Such a shift in pore size distribution is generally considered beneficial for the strength of cement-based materials [44]. After 200 d of exposure, the mesopore peak of both mixtures shifts distinctly toward larger pore sizes. This alteration negatively influences the strength and durability of the HFRC matrix [45]. Additionally, both exhibit a prominent peak at 20,000 nm, indicating that the microstructure has coarsened, a trend also observable in Figure 10b, where the volume of large capillary pores increases across the entire range of pore diameters with prolonged salt spray exposure.

The data presented in Figure 10b offers a clearer insight into the impact of salt spray exposure on the pore structure of HFRC mixtures. At 0 d of exposure, with the incorporation of PP fibers, the mesopore fraction of the HFRC mixtures increases from 34.22% in M0 to 38.51% in HF2. This suggests that the addition of PP fibers to HFRC results in a finer pore size distribution, which may, in turn, positively influence the strength development of the HFRC. After 200 d of exposure, a reduction in the mesopore and middle capillary pore fractions is observed in both mixtures, while the fraction of large capillary pores significantly increases. This observation provides a clear explanation for the coarsening of the pore structure in HFRC mixtures after long-term salt spray exposure, which is a contributing factor to the reduction in compressive strength. Notably, the large capillary pore fraction in HF2 (62.12%) after 200 d is lower than that in M0 (72.16%), indicating that the bridging effect of PP fibers can effectively suppress the further development and connectivity of pores caused by chloride corrosion, thereby mitigating the deterioration of the HFRC pore structure.

### 3.2. Ultrasonic Wave Test Results of HFRC

**Figure 11:**
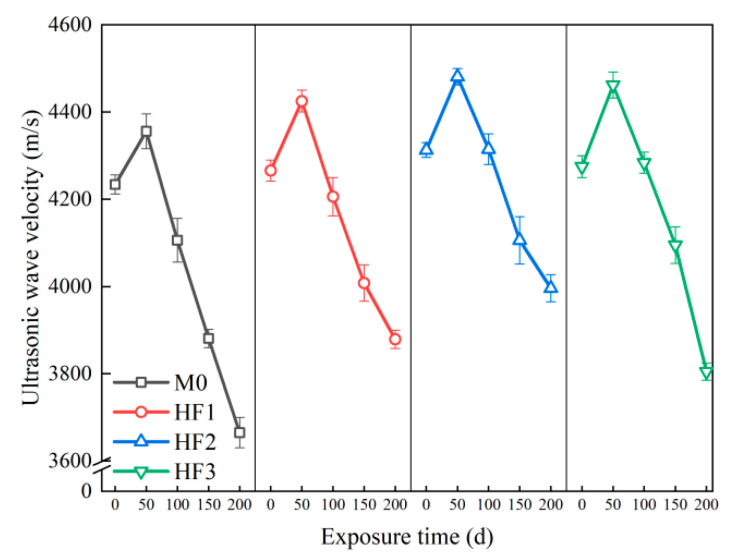
Ultrasonic wave velocity of HFRC mixtures with different exposure times.

Figure 11 reveals the ultrasonic wave velocity of HFRC mixtures subjected to salt spray conditions over varying exposure times. At 0 d, the ultrasonic wave velocity of all four HFRC mixtures ranged between 4234 m/s and 4313 m/s. At this point, the concrete had not been subjected to chloride-induced corrosion, and its microstructure remained relatively intact, resulting in high stability and consistency of the wave velocity. At 50 d, the wave velocity of M0 increased to 4356 m/s, reflecting a 2.88% increase compared to 0 d, showing a trend similar to that observed in the early-stage development of compressive strength. After 100 d of exposure, the wave velocity of M0 decreased to 4106 m/s, marking a decline from the value at 0 d. As the exposure time continued, the wave velocity at 150 and 200 d dropped to 3871 m/s and 3665 m/s, respectively, representing reductions of 8.57% and 13.44% relative to the original value. This suggests that M0, containing only steel fibers, experienced a significant deterioration in the matrix’s compactness under long-term salt spray exposure, leading to a marked reduction in ultrasonic wave velocity.

On the other hand, for the HFRC mixtures with incorporating PP fibers, the ultrasonic wave velocity of the specimens after 50 d of exposure was higher compared to the control group (M0). Among them, HF2 exhibited the highest wave velocity, reaching 4481 m/s, which is 2.87% greater than that of M0. The enhanced compactness of the HFRC matrix can be explained as follows: In the preliminary stages of chloride corrosion, the compensatory effects of the hydration reaction significantly outweigh the damage induced by chloride penetration [46]; moreover, the PP fibers facilitate the refinement of the pore structure, thereby enhancing the compactness of the microstructure [43]. Nevertheless, at 100 d, a decrease in wave velocity was observed; HF2 maintained a relatively high wave velocity (4315 m/s), comparable to its original value (4313 m/s). HF1 and HF3 exhibited wave velocities of 4206 m/s and 4284 m/s, respectively. At 150 d, the rate of decline became more pronounced, with the wave velocity of HF1 dropping to 4008 m/s, HF2 to 4106 m/s, and HF3 to 4095 m/s, indicating the escalating impact of chloride corrosion and its increasing damage to the matrix compactness. Ultimately, at 200 d, a sharp decrease in wave velocity was observed for all HFRC mixtures, with HF1 and HF2 dropping to 3879 m/s and 3996 m/s, respectively, while HF3 experienced the most severe decline, reaching 3805 m/s. However, the final ultrasonic wave velocities of the HF3 remained higher than those of M0 (3665 m/s). Therefore, the incorporation of PP fibers into HFRC mixtures significantly enhances the compactness of the matrix and improves the resistance to salt spray corrosion.

**Figure 12:**
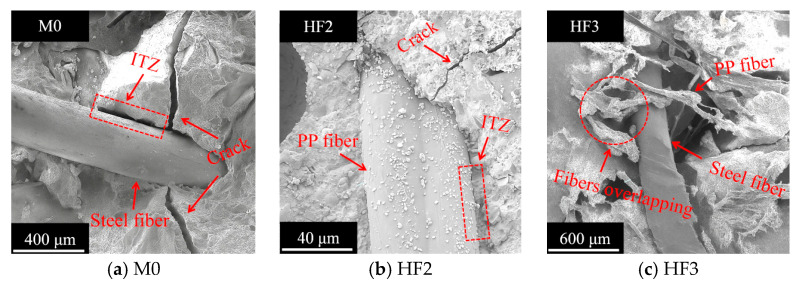
Representative images of the fiber/matrix interface.

It should be noted that the compactness of the matrix is closely associated with the degree of bonding at the fiber-matrix interfacial transition zone (ITZ). Therefore, by analyzing the morphology of the fiber/matrix interface, the influence of fiber type and content on the matrix compactness can be accurately assessed. Figure 12 illustrates the fiber/matrix interface in HFRC mixtures.

As depicted in Figure 12a, the ability of steel fibers to restrain the development of microcracks in the matrix is relatively weak. Owing to the continuous accumulation of chloride, a penetrating crack forms at the fiber/matrix interface of M0. The presence of these penetrating cracks accelerates the chloride penetration process, leading to a decrease in the matrix’s compactness [47].

In contrast, as observed in Figure 12b, the microcracks within the matrix are interrupted upon contacting PP fibers. Furthermore, compared to the steel fiber-matrix ITZ thickness in Figure 12a, the thinner ITZ thickness of the PP fiber-matrix indicates a strong bond between the PP fibers and the matrix [48]. This synergistic effect prevents chloride ions from penetrating deeply and eroding the interior of the matrix, thereby enhancing the concrete’s resistance to chloride corrosion.

However, if the PP fiber content is excessive, the expected crack-interrupting effect is typically not achieved. As shown in Figure 12c, due to the incorporation of 0.5% by volume of PP fibers in HF3, uneven dispersion of the fibers within the matrix is observed, with fibers overlapping and interlocking. As previously reported in Ref. [12], such uneven dispersion inhibits the cement paste from fully filling the voids between the fibers and the matrix. As a result, cracks can easily pass through the overlapping fibers, thereby allowing more chloride ions to penetrate the matrix. The aforementioned cause provides a reasonable explanation for the most severe decline in wave velocity of HF3 after 200 d of exposure, compared to HF1 and HF2.

In conclusion, salt spray conditions result in the continuous generation and propagation of cracks within concrete. PP fibers can restrain the development of these cracks, thereby delaying the degradation of concrete durability [49]. This is the primary reason that HFRC mixtures incorporating PP fibers exhibit significantly enhanced resistance to chloride penetration compared to SFRC.

### 3.3. Chloride Content Test Results of HFRC Mixtures

**Figure 13:**
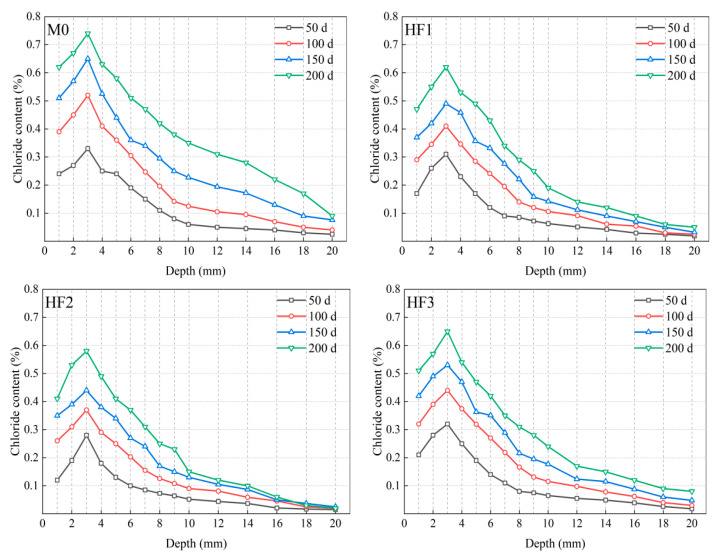
Chloride content distribution of HFRC mixtures with different exposure times.

The distribution of the chloride content in HFRC under different salt spray exposure times is displayed in Figure 13. These curves allow for the extraction of key chloride corrosion parameters, such as peak chloride content and critical corrosion depth. After exposure for 50, 100, 150, and 200 d, the chloride content distribution curves of all HFRC mixtures exhibit two distinct development stages: (1) the ascending stage in the convection zone, where chloride ions, transported by the salt spray, accumulate on the concrete surface. Subsequently, chloride ions diffuse from the surface to deeper layers via capillary absorption and convective processes, with the concentration progressively increasing with depth, ultimately attaining a maximum value at a defined depth [50]. This maximum concentration is defined as the peak chloride content. It can be observed that, in this experiment, the depth of the convection zone for each mixture remained consistent at 3 mm; (2) the descending stage in the diffusion zone, where chloride ions diffuse from regions of higher concentration to regions of lower concentration, resulting in a gradual decrease in the concentration with increasing depth. Once the chloride concentration decreases below 0.1%, the diffusion force for chloride ions is substantially reduced, thereby minimizing the threat to the concrete’s durability and structural stability [51]. The depth at which the chloride concentration decreases to 0.1% for the first time is designated as the critical corrosion depth. All chloride corrosion parameters for each HFRC mixture are summarized in Table 4.

**Figure 14:**
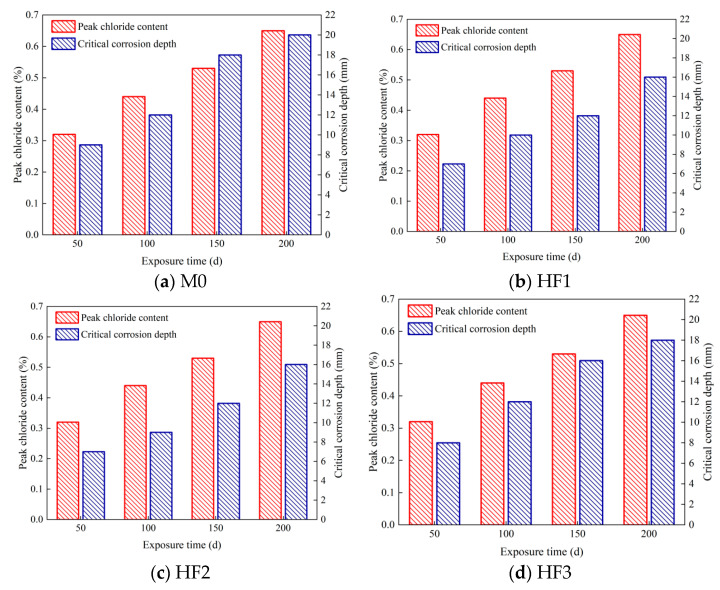
Chloride corrosion parameters of HFRC mixtures.

Figure 14 provides a comprehensive analysis of the chloride corrosion parameters, underscoring the significant differences among all HFRC mixtures. As depicted in Figure 14 and Table 4, after 50 d of exposure, the variation in peak chloride content among the HFRC mixtures was minimal, with values ranging between 0.28% and 0.33%. This can be ascribed to the fact that, during the early stages of corrosion, chloride ion penetration primarily occurs via salt spray deposition and capillary absorption at the concrete surface. At this stage, the presence of fibers has a relatively minor effect on surface adsorption. As exposure time increases, the peak chloride content of each HFRC mixture continues to rise. Specifically, for M0, the peak chloride content increased from 0.33% at 50 d to 0.52% at 100 d, and further to 0.65% at 150 d, reflecting increments of 57.58% and 96.97%, respectively. This progression is attributed to the gradual diffusion of chloride ions into the concrete matrix, which leads to a reduction in internal alkalinity, subsequently altering the mechanical properties of the cementitious matrix [52]. Furthermore, this process promotes the formation of additional defects in the fiber-matrix ITZ, further compromising the stability of the matrix. The weakened matrix, characterized by reduced strength and stability, facilitates enhanced chloride ion penetration, thereby driving a continued increase in chloride content. Ultimately, at 200 d, the peak chloride content for M0 reached 0.74%, moderately exceeding the 0.7% threshold reported for high-strength concrete in Thomas’s study [53], indicating the onset of an irreversible corrosion stage, during which the concrete’s durability is severely damaged.

In contrast, the increase in peak chloride content for the HF series was comparatively lower. For instance, the peak chloride content for HF2 at 100, 150, and 200 d was 0.37%, 0.44%, and 0.58%, respectively, all of which were lower than those observed for M0 at the same exposure time. Similarly, the peak chloride content for HF1 and HF3 at 200 d was 0.62% and 0.65%, respectively, representing reductions of 16.22% and 12.16% compared to M0. These findings suggest that the incorporation of PP fibers into the HFRC mixtures effectively reduced chloride contents and improved the concrete’s resistance to chloride penetration.

As presented in Figure 14 and Table 4, the variation in the critical corrosion depth among the HFRC mixtures closely mirrors the trend observed for the peak chloride content. As exposure time increases, the critical corrosion depth of M0 increased from 9 mm at 50 d to 12 mm at 100 d, and reached 18 mm at 150 d, with the rate of corrosion deepening accelerating progressively. Notably, after 200 d of exposure, the critical corrosion depth of M0 reached 20 mm, which represents the upper limit of the measurement range for this experiment; the actual corrosion depth may exceed this value. Such an extensive degree of corrosion presents a significant threat to both the performance and structural stability of the concrete [54].

**Figure 15:**
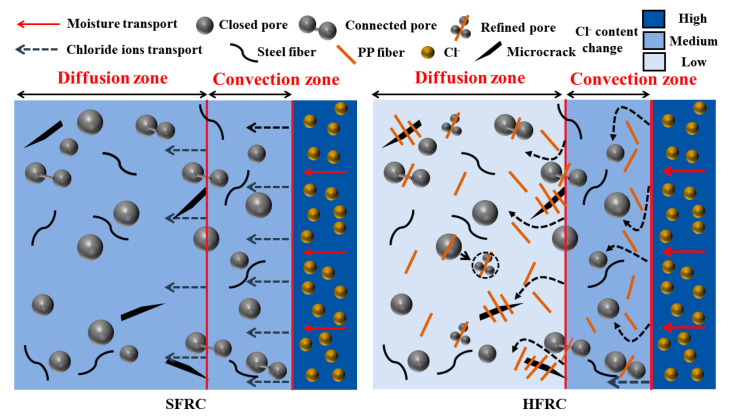
Diagram of chloride penetration of HFRC mixtures under salt spray conditions.

For the HF series, while the critical corrosion depths of HF1, HF2, and HF3 steadily increased with exposure time, they consistently remained below that of M0 across all exposure times. For example, at 50 d, the critical corrosion depths of HF1 and HF2 were both 7 mm, 2 mm lower than that of M0. At 200 d, the critical corrosion depths for HF1, HF2, and HF3 were 16 mm, 16 mm, and 18 mm, respectively, all remaining within the measurement range of the experiment. Figure 15 depicts the chloride penetration process within the HFRC mixtures under the salt spray conditions. As observed, the presence of PP fibers appears to create a convoluted path for chloride penetration, which may reduce the permeability of chloride ions to some extent. On the other hand, PP fibers refine the pore structure and restrict pore connectivity, leading to the formation of a more convoluted and discontinuous pore network, which in turn reduces the transmission pathways for chloride ions [12,55]. Therefore, the presence of PP fibers leads to a corresponding reduction in the critical corrosion depth of the HFRC mixtures.

**Figure 16:**
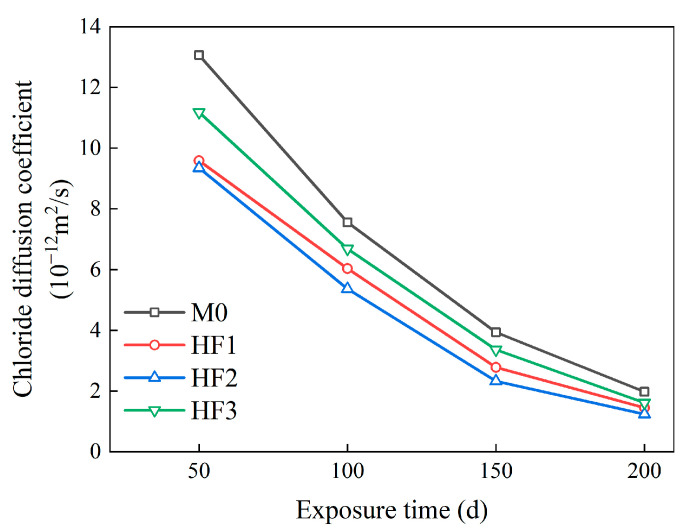
Chloride diffusion coefficients of HFRC mixtures at different exposure times.

It is crucial to underscore that both the peak chloride content and the critical corrosion depth are intrinsically linked to the rate of chloride penetration within the concrete matrix [56]. Therefore, investigating the chloride diffusion coefficient can provide enhanced insights into the influence of fiber type and content on the concrete’s resistance to chloride corrosion. Based on Fick’s second law [57], under salt spray conditions, the depth of the convection zone induced by chloride penetration is significantly smaller than the thickness of the diffusion zone. By excluding chloride penetration in the convective zone, the modified form of Fick’s second law is expressed in Equation (1). The data from Figure 13 is applied, with the peak chloride content set to zero, and a fitting procedure is applied to the chloride content within the diffusion zone. The calculated chloride diffusion coefficient is shown in Figure 16.

(1) $$C\left(x,t\right)=C_{0}\times \left(1-\frac{x}{2\sqrt{t\times D_{t}}}\right)$$

where *C*(*x*,*t*) represents the chloride content at a specific corrosion depth *x* and exposure time *t* under salt spray conditions; *C*_0_ is the initial chloride content, set as the peak chloride content, in accordance with the recommendations of Andrade et al. [58]; *D_t_* is the chloride diffusion coefficient.

As illustrated in Figure 16, with the exposure time increasing from 50 to 200 d, the chloride diffusion coefficient decreases, which contrasts with the observed trends in peak chloride content and critical corrosion depth. At 100 d of exposure, the chloride diffusion coefficient of M0 was measured at 7.56 × 10^−12^ m^2^/s, indicating a marked reduction from the original value of 13.07 × 10^−12^ m^2^/s at 50 d. As the exposure time increased to 150 d, the chloride diffusion coefficient further decreased to 3.94 × 10^−12^ m^2^/s; at 200 d, it reached a final value of 1.98 × 10^−12^ m^2^/s. This trend can be attributed to the progressive accumulation of chloride precipitates, which progressively obstruct the diffusion pathways as the exposure time increases [13]. Consequently, this results in a more concentrated accumulation of chlorides within the cementitious matrix, thereby accelerating the corrosion process of the concrete and driving the continuous increase in both peak chloride content and critical corrosion depth.

In contrast to M0, after 50 d of exposure, the chloride diffusion coefficients of HF1, HF2, and HF3 were 9.59 × 10^−12^ m^2^/s, 9.35 × 10^−12^ m^2^/s, and 11.19 × 10^−12^ m^2^/s, respectively, reflecting reductions of 26.63%, 28.46%, and 14.38%. The reduced chloride diffusion coefficients in the HF series can be attributed to the crack-inhibiting effect created by the PP fibers, which restricts the formation of microcracks [59,60]. As a result, the overall chloride ion transport and permeability are reduced. This is also the reason for the reduced peak chloride content and critical corrosion depth observed in HFRC mixtures incorporating PP fibers.

After 200 d of salt spray conditions, the chloride diffusion coefficient of HF2, containing 0.3 vol% PP fibers, was the lowest among the HF series, measuring only 1.24 × 10^−12^ m^2^/s. In contrast, the chloride diffusion coefficient of HF3, with 0.5 vol% PP fiber, reached the highest value (1.62 × 10^−12^ m^2^/s). These findings suggest that an excess of PP fibers may weaken the chloride penetration resistance of HFRC mixtures, emphasizing the need for strict control over the volume content of PP fibers. In conclusion, this study suggests that incorporating 0.3 vol% PP fibers is the most effective approach among the tested contents to enhance the salt spray corrosion resistance of HFRC mixtures.

## 4. Conclusions

This study presents a novel approach that incorporates polypropylene (PP) fibers to partially replace steel fibers in the formulation of hybrid fiber reinforced concrete (HFRC), thereby enhancing its durability in a salt spray environment. The principal findings can be summarized as follows:

(1) The HFRC mixtures prepared in this study exhibit a compressive strength of approximately 65 MPa at 28 d. After 50 d of salt spray exposure, this value increases slightly. However, when exposed for over 100 d, the compressive strength of all mixtures undergoes a significant decline. Notably, the decline in compressive strength of the HFRC mixtures containing PP fibers is significantly slower than that of the M0 mixtures, which incorporate only steel fibers, and after 200 d of exposure, the former still qualifies as high-strength concrete (>60 MPa). Microstructural analysis indicates that PP fibers effectively mitigate the further expansion and connectivity of pores induced by chloride corrosion, a mechanism that substantially slows the degradation of compressive strength.

(2) After 50 d of exposure, the ultrasonic wave velocity of all four HFRC mixtures was slightly higher than that at 0 d. However, after exposure for 100 and 150 d, the wave velocity of the samples rapidly decreased, indicating a significant deterioration in the compactness of the matrix. Despite the substantial decrease in wave velocity, the ultrasonic velocity of the HF series after 200 d of exposure remained higher than that of the control group (M0); this can be attributed to the strong bonding between the PP fibers and the matrix, which effectively prevents the propagation of microcracks induced by chloride corrosion.

(3) The incorporation of flexible PP fibers significantly enhances the chloride resistance of HFRC mixtures. For instance, after 200 d of exposure, the addition of 0.3 vol% PP fibers reduced the peak chloride content and critical corrosion depth of the HFRC mixtures to 0.58% and 16 mm, respectively, from the values of M0 (0.74% and 20 mm). The presence of PP fibers introduces a tortuous pathway for chloride ions, thereby improving the material’s resistance to chloride corrosion. Moreover, the chloride diffusion coefficient calculations reveal that the HF2 mixture exhibits the lowest value across all exposure times, indicating that incorporating 0.3 vol% PP fibers is the most effective strategy to enhance the chloride corrosion resistance of HFRC mixtures.

In conclusion, this study primarily investigated the effects of PP fibers on the mechanical properties and chloride penetration resistance of HFRC mixtures. The results reveal that PP fibers not only improve the pore structure and mechanical properties, but also enhance the resistance to chloride penetration. Future research should build upon these findings to explore related areas further. Key areas of investigation will include exploring a wider range of fiber contents to identify the optimal fiber content for improving durability. Additionally, to address the lack of long-term field validation, HFRC mixtures will be exposed to actual marine conditions, enabling a direct comparison with the laboratory data presented here. Moreover, while the chloride diffusion coefficient was estimated using fitting formulas, future work will employ the rapid chloride migration (RCM) method to conduct more precise analyses of how PP fibers impact chloride ion diffusion resistance. These efforts will contribute to a more comprehensive understanding of the material’s performance and its potential for applications in marine engineering.

## Figures and Tables

**Table 1:** Chemical compositions of binder materials.

	SiO_2_	Al_2_O_3_	CaO	Fe_2_O_3_	MgO	MnO	K_2_O	TiO_2_
C	28.5%	11.5%	48.9%	4.9%	3.0%	0.4%	1.6%	1.2%
FA	48.8%	29.9%	9.4%	6.9%	1.9%	0.2%	1.5%	1.4%
SF	>85%	--	--	--	--	--	--	--

**Table 2:** The physical and mechanical properties of steel fiber and PP fiber.

	Length (mm)	Diameter (mm)	Elastic Modulus (GPa)	Tensile Strength (MPa)	Density (kg/m^3^)	Elongation
Steel fiber	40	0.30	200	1270	7800	5%
PP fiber	12	0.03	4.8	500	910	17%

**Table 3:** HFRC mix proportions (kg/m^3^).

	C	FA	SF	CA	SS	W	SP	Volume Fraction	
								Steel Fiber	PP Fiber
M0	400	100	635	1165	27	169	4	2.0%	0
HF1	400	100	635	1165	27	169	4	1.9%	0.1%
HF2	400	100	635	1165	27	169	4	1.7%	0.3%
HF3	400	100	635	1165	27	169	4	1.5%	0.5%

**Table 4:** Summarized chloride corrosion parameters of HFRC after salt spray exposures.

Mixture	Exposure Time (d)	Peak Chloride Content (%)	Critical Corrosion Depth (mm)
M0	50	0.33	9
	100	0.52	12
	150	0.65	18
	200	0.74	20
HF1	50	0.31	7
	100	0.41	10
	150	0.49	12
	200	0.62	16
HF2	50	0.28	7
	100	0.37	9
	150	0.44	12
	200	0.58	16
HF3	50	0.32	8
	100	0.44	12
	150	0.53	16
	200	0.65	18

## Data Availability

The original contributions presented in this study are included in the article. Further inquiries can be directed to the corresponding author.
